# Neural Plasticity following Abacus Training in Humans: A Review and Future Directions

**DOI:** 10.1155/2016/1213723

**Published:** 2016-01-04

**Authors:** Yongxin Li, Feiyan Chen, Wenhua Huang

**Affiliations:** ^1^Institute of Clinical Anatomy, School of Basic Medical Sciences, Southern Medical University, Guangzhou 510515, China; ^2^Bio-X Laboratory, Department of Physics, Zhejiang University, Hangzhou 310027, China

## Abstract

The human brain has an enormous capacity to adapt to a broad variety of environmental demands. Previous studies in the field of abacus training have shown that this training can induce specific changes in the brain. However, the neural mechanism underlying these changes remains elusive. Here, we reviewed the behavioral and imaging findings of comparisons between abacus experts and average control subjects and focused on changes in activation patterns and changes in brain structure. Finally, we noted the limitations and the future directions of this field. We concluded that although current studies have provided us with information about the mechanisms of abacus training, more research on abacus training is needed to understand its neural impact.

## 1. Introduction

The brain is the source of behavior but is in turn modified by the behaviors it produces, such as when acquiring and practicing particular skills. In recent decades, a steadily growing number of studies in human and animals have confirmed the notion that learning new skills can cause functional and structural reorganization of the brain [1, 2]. Recently, abacus-based mental calculation (AMC) training, a specific method to gain arithmetic skills, has received much attention [3–8].

The abacus is a sort of traditional calculator that has been used in China, Korea, Japan, and India since 1200 AD for rapid precise calculations [9]. The abacus can be used to perform arithmetic, including addition, subtraction, multiplication, division, and root calculations. It represents numbers via an arrangement of beads in columns, where each column represents a place value that increases from right to left [10]. AMC experts are able to perform most arithmetic operations not only with physical abacus but also with abacus mental calculations, with unusual speed. This skill can be acquired in several steps with long-term training. First, abacus players learn to operate abacus beads in a corresponding apparatus with both hands. Second, they learn to imagine and operate abacus beads in their minds. As their calculation skills improve, they can manipulate numbers via an imagined abacus without actual finger movements. Usually, AMC experts have the ability to mentally calculate numbers of 10 digits or longer with unusual speed and accuracy [11, 12]. Psychological [10, 13–15] and neuroimaging [3, 4, 7] studies have shown that the brain activation patterns of AMC experts are different from those of nonexperts and that calculation skills were enhanced significantly after abacus training compared with average control subjects. Although the effects of AMC training are well known, the underlying neural mechanism of these effects remains elusive.

In this review, we systematically reviewed the published works comparing abacus experts and average control subjects to illustrate and discuss the neural mechanism underlying AMC training. First, we highlighted recent achievements in AMC training, including relevant findings from behavioral and neuroimaging studies. Second, we concluded with some critical considerations and directions for future research in this field.

## 2. Behavioral Findings of AMC Training

Previous behavioral studies have shown that AMC experts may calculate with a “mental abacus” and that such a calculation method is different from the exact mental calculation method used by nonexperts [10]. Visuospatial strategies are used by abacus experts for digit memory and mental calculation tasks [11, 13–17], whereas linguistic strategy played an essential role in these processing tasks in nonexperts [18, 19]. For example, Hatano et al. [13] investigated the processing mechanism of AMC experts during mental calculations. In this study, AMC experts were given various restrictions and distractions during the addition of ten numbers of 3–5 figures. As expected, the AMC experts could answer simple nonmathematical questions during abacus calculation without increasing time or errors, but answering extraneous mathematical questions was very difficult. This study suggests that AMC experts apply two different operation strategies during mental calculation tasks and nonmathematical tasks. Two similar studies by this research group on abacus training were carried out [16, 20]. The digit-span capacity of three national champions in mental calculation was measured while given various memory tasks [16]. The experts had a much larger digit span than the controls, but their span for letters of the alphabet or fruit names showed no difference. Their digit memory was disrupted more by concurrent visual-spatial tasks than by aural-verbal tasks, whereas their letter memory was disrupted more by concurrent aural-verbal tasks than by visual-spatial tasks. These results suggest that these experts represent digits but not alphabet letters or other verbal items in the form of visuospatial images. The experts seemed to have a “mental abacus” to visuospatially represent a number composed of many digits. Abacus experts mainly use a visuospatial strategy to process numerical information. This relationship was also observed between the representational changes in digit memory and the extent of expertise in mental abacus operation [20]. Five groups of operators differing in AMC expertise level were recruited to perform a digit reproduction task. A simple aural-verbal or visual-spatial task was interpolated between the presentation and reproduction of the digits. The research showed that the digit memory of more skilled operators was less vulnerable to an aural-verbal interpolated task and more vulnerable to visual-spatial tasks. The above three studies provided evidence that different strategies were used in the processing of numerical information between AMC experts and nonexperts. Subsequent studies have also examined how AMC experts process imagery [14] and reported that they use a “mental abacus” to represent numbers [11, 15]. Based on the above evidence, we can say that long-term AMC training may have some effect on an individual's mental calculation skills [11, 13] and conceptual knowledge of the numeration system [15]. Different strategies used in the processing of numerical information may lead to differences in digit memory span, reaction time, and accuracy between AMC experts and nonexperts [13, 14, 20]. AMC experts mainly rely on visual/visuospatial strategies for the processing of numerical information, whereas nonexperts mainly rely on linguistic strategies.

Before functional magnetic resonance imaging (fMRI) technology, behavior experiments were designed to test the hypothesis that AMC experts are mainly dependent on the right hemisphere of the brain for mental calculation processing, whereas nonexperts are mainly dependent on the left hemisphere [17, 21]. One study examined the effects of presented mental calculations and word-reading tasks on sequential finger tapping [21]. For aurally presented mental calculations, AMC experts showed greater interference effects on left hand tapping, whereas the controls showed greater interference effects on right hand tapping. For visually presented mental calculations, AMC experts showed a nonsignificant tendency towards greater interference in the left hand, whereas the controls showed no hand difference. For word-reading, both groups showed greater interference in the right hand than in the left hand. These results revealed that AMC experience may affect brain activation patterns. The right hemisphere of the AMC experts engages in mental calculation, whereas the left hemisphere contributes to mental calculations in nonexperts. This work is in accordance with the studies by Hatano et al. [16, 20] and confirmed the view that different strategies were used in the processing of numerical information between AMC experts and nonexperts. The effects of AMC training on imagery processing ability were also discussed based on visual field differences between the AMC experts and the controls [17]. Multicondition matching tasks (word, picture, digit sequence, and abacus-picture) were designed to examine visual processing ability. All materials were presented to either the left or the right visual field. In all conditions, the AMC experts performed faster than the controls and showed a left visual field advantage under imagery conditions, whereas the controls showed a right visual field advantage under rehearsal conditions. Thus, AMC training may have some effect on individual visual processing ability. However, a common confounding factor is present in all behavioral studies of abacus training. Preexisting differences may be a factor that interferes with research results. The reported differences between the two groups may have been present prior to abacus training. The trained individuals may come to the table with advanced visuospatial abilities that predisposed them to seek out and excel at AMC training. Studies that perform examinations before AMC training would allow for the opportunity to eliminate this interference. Another way to solve this problem is to conduct a double-blinded and placebo-controlled study, which should address the problem of group division and allow for specific comparisons between groups to reveal the mechanisms underlying AMC training.

During the learning process, cognitive skills are used to assimilate, organize, and process information. Previous studies have found that cognitive training has an effect not only on skills that are trained, but also on skills that are not explicitly trained [22, 23]. As special cognitive training, a transfer effect of abacus training has been found in the domains of other cognitive functions, such as general intelligence [24] and memory [9]. In an early study by Stigler et al. [12], AMC skills were found to develop primarily as a result of practice rather than socioeconomic status, ability, or previous mathematical knowledge. It was also found that AMC skills may contribute to cognitive development and future achievement. Recently, a study focused on the effects of AMC training on mathematical achievement in children with mild mental retardation and confirmed the idea that AMC skills contribute to general cognition, showing that AMC training has positive effects on student learning [25]. Abacus operation and mental abacus calculation are imbedded in all aspects of a math curriculum. The results showed that the experimental group outperformed the control group in most skill areas, including computation skills, mathematics concepts, and the application of math skills to real-life situations. This study supports the development of an innovative strategy in math curriculum reform and for training students with mathematical learning disabilities. The effects of AMC training on intelligence were also investigated in a large sample of children between 7 and 11 years in Sudan [24]. AMC training was performed two hours a week for 34 weeks. At the end of the training, the standard progressive matrices performance was enhanced significantly in the trained group. This result suggests that problem-solving skills acquired through AMC training may increase intelligence. A recent behavioral study by Wang et al. [8] examined the question of whether the extraordinary gains of abacus experts in mental arithmetical ability were accompanied by an improvement in numerical processing efficiency. A numerical Stroop paradigm was used to examine the numerical processing efficiency of experienced mental abacus children, mental abacus beginners, and their respective peers. Experienced abacus children were less influenced than their peers by physical size information when intentionally processing numerical magnitude information, but they were more influenced than their peers by numerical magnitude information when intentionally processing physical size information. No differences were found between the abacus beginners and their peers in the same conditions. These findings indicated that improvement in numerical processing efficiency could be achieved through long-term intensive AMC training. The effect of abacus training on attention in children has also been studied [26]. Elementary school children were recruited, and their attention, memory, and arithmetic abilities were measured. AMC children performed significantly better in number memory and calculation. On the attention test, trained children committed fewer commission errors in tasks requiring sustained attention inhibition and selective inference attention. Fewer commission errors suggested that the trained children exhibited better response inhibition. This work suggests that abacus training may be an option for children with cognitive problems. Considering the effects of AMC training on mental calculation, general intelligence, memory ability, and numerical processing efficiency, we conclude that abacus skills may contribute to cognitive development. AMC training may have tremendous application value for children with math learning disabilities and people seeking to enhance their intelligence and cognitive memory skills.

Based on the above evidence, the consequences of developing AMC skills were reviewed. Long-term AMC training was found to affect both calculation skills and conceptual knowledge of the numeration system. This training may result in qualitative changes in a child's ability to represent numerical information through the development of a “mental abacus” and improve response inhibition. Furthermore, AMC skills can be used to develop number concepts, increase efficiency in mathematical calculations, achieve substantial gains in numerical processing efficiency [8], and improve the ability of students to apply mathematical skills to real-life situations [9, 24, 25]. Most of these behavioral studies were performed on children, and the brains of children can develop quickly. The cross-sectional design of these studies cannot exclude the effect of brain development. Future studies with a double-blinded, placebo-controlled, or longitudinal design would eliminate these confounding factors and better evaluate the functional behavioral relevance of AMC training.

## 3. Neuroimaging Findings of AMC Training

Behavioral studies have shown that AMC training affects both calculation skills and number concepts. However, the neural mechanism of this skill cannot be explained by behavioral effects alone. Neuroimaging data can be used as an important outcome measure [27]. As such, these data have the potential to reveal insights about cognitive processes, which are not attainable using behavioral studies alone. Imaging technologies such as positron emission tomography (PET) and fMRI offer the opportunity to investigate human brain organization* in vivo* noninvasively [28, 29]. In each of these techniques, changes in image intensity with differences in cognitive state can provide information about changes in the underlying physiologic state of the brain [30]. The application of neuroimaging technology in individuals with AMC training has identified related differences in the brain [3, 4] and provided new evidence for brain plasticity [1].

PET is a general name for techniques that rely on the introduction of radioactive compounds into body tissue (including brain) that may be used to assess local metabolism. The effects of long-term AMC training on brain activity were studied with this technology [31, 32]. Different regional cerebral blood flow patterns using ^15^o-water PET were compared between AMC experts and controls while these subjects were performing mental calculation tasks. The visuospatial network, including the bilateral parietal/frontal areas, was involved in addition tasks for the AMC experts. The language network, which includes the inferior frontal areas, was observed in the controls. The first application of fMRI to the field of AMC training was designed by Tanaka et al. [7]. In this study, a digit delayed match-to-sample task was designed to examine adult brain activity. Cortical areas related to visuospatial working memory, including the bilateral superior frontal sulcus and superior parietal lobule (SPL), were significantly activated in adult AMC experts. In the controls, activity was greater in the cortical areas related to verbal working memory, including Broca's area. These findings were consistent with those of previous behavioral studies [16, 33]. It is possible that a spatial representation of numbers is achieved through long-term abacus training. During the digit delayed match-to-sample task, both groups may use different strategies. These results provide the first neurophysiological evidence that abacus experts utilize visuospatial representations for digit memory. Subsequently, improved match-to-sample tasks (digit and bead materials) were used to study long-term abacus training in children [6]. Compared with the average control subjects, the abacus-trained children showed higher activity in the right posterior superior parietal lobule/superior occipital gyrus and the right supplementary motor area in both tasks. This activated pattern was consistent with the findings of Tanaka et al. [7] and further supported the important role of the frontoparietal network in digit working memory in AMC experts. Moreover, a very important study by Hanakawa et al. [4] provided direct evidence that mental calculations by adult AMC experts primarily depended on brain areas involved in visuospatial imagination. The neural correlates associated with three mental-operation tasks (numeral, spatial, and verbal) were compared among experts and controls. The right frontoparietal areas, especially the posterior superior parietal cortex, were significantly activated in the AMC experts compared with the controls during numeral mental-operation tasks. These differences may indicate that more visuospatial/visuomotor imagery processes are involved in mental calculation for AMC experts compared with nonexperts. The neural correlates of mental calculation were also explored in children with AMC training [3]. Simple and complex serial calculation tasks were designed, and the activated patterns found were quite different between the two groups. In the experts, significant activation was mainly observed in the frontotemporal circuit during simple addition and the frontoparietal circuit during complex addition. In the controls, both tasks exhibited similar activation patterns consisting of significant increases in the bilateral inferior parietal lobes and the prefrontal and premotor cortices. The works by Hanakawa et al. and Chen et al. separately indicated that mental calculation processing in adult and child experts primarily depended on the brain areas involved in visuospatial imagination rather than those involved in linguistic information processing [3, 4]. The difference in brain activity between the AMC and the controls in both studies was a result of a different weight of calculation strategy. A visuospatial strategy was more involved during mental calculation for the abacus experts, whereas a linguistic strategy was more involved during mental calculation for the nonexperts. Several studies have used fMRI technology to examine the neural correlates underlying abacus mental calculation in adults [4, 34, 35] and children [3, 36, 37]. Digit memory and mental calculation in the abacus-trained subjects were associated with the enhanced involvement of neural resources for visuospatial information processing [3, 4, 6, 7]. These neuroimaging findings consistently revealed that abacus-trained subjects are more dependent on the right frontoparietal network for mental calculations.

However, the conclusions of the functional neuroimaging studies are based entirely on a comparison between an expert group and a native group. This type of design is used due to ethical concerns or educational restrictions. It is difficult to recruit a proper control group from a training group. As a result, the neural mechanism is simply inferred from observed brain activation. Although the difference in brain activation can be explained by the use of different strategies by the AMC experts and the controls, individual differences have not been considered. The effects of individual differences were not well controlled in the above neuroimaging research, which may have affected the statistical results. Different individuals may have different strategic preferences during the same cognitive processes. Some nonexperts may also use a spatial strategy during mental calculation. In this case, the differences in brain activation between the groups can be explained by the idea that AMC experts may have developed good spatial skills after long-term abacus training. Future experiments should be designed to identify the strategy used by nonexperts during mental calculation. When we classify the nonexperts using different strategies for brain activation comparisons during mental calculation with AMC experts, more accurate and comprehensive results will be achieved. Based on these results, the neural mechanism would be explored. Furthermore, the link between the results of neural studies and behavioral studies is not well established, as there is a separation between behavioral and neuroimaging research. To establish a link, neural imaging must be collected with additional intervening data such as localizer tasks, brain-behavioral correlations, longitudinal studies, or some combination thereof. For example, behavioral experiments designed by Hatta and Ikeda [21] were performed to infer the neural mechanisms used by abacus experts. The results showed that different strategies between the AMC experts and the nonexperts were being used to process numerical information. Such a conclusion cannot be rigorously established using behavioral data alone. With the development of imaging technology, we suggest that fMRI data should be collected along with behavioral data, thereby allowing for the acquisition of more relevant information such as the brain activation, behavioral performance, and brain activation-behavioral correlations. For most neuroimaging studies, the sample sizes are relatively small. With the development of imaging techniques and statistical analyses, sample sizes have increased. However, future studies with larger sample sizes are still required.

Recently, a longitudinal fMRI study of a skilled abacus user with a right hemispheric brain lesion identified the importance of the right frontoparietal circuit for performing AMC [35]. The patient was a 57-year-old left-handed female. She began her abacus training at an abacus school when she was a child and trained in physical and mental abacus operation for 3 years. After training, she kept using abacus-based mental calculations and mnemonic strategies in everyday activities and did not lose her ability. In July of 2009, she suffered a right hemispheric infarct in the territory of the anterior and middle cerebral arteries. Two months after the stroke, a clinical neuropsychological evaluation was administered. She noticed that she could not use a mental abacus strategy for the digit-span test. She was not able to generate vivid mental imagery of an abacus as the image of her abacus was very fragile. Six months after her stroke, it remained difficult for her to generate a visual image of a mental abacus. The first fMRI session was conducted at that time. The results showed more brain activity during mental calculation at 6 months after her stroke in Broca's areas, the left dorsolateral prefrontal area, and the inferior parietal lobule. Thirteen months after her stroke, she reported that her capacity for visual imagery of a mental abacus had recovered and that she was able to shift the calculation strategy from linguistic to visuospatial. At that time, a second fMRI session was conducted. During mental calculation, more brain activity was observed in the left superior parietal lobule, indicating that her brain activity had in fact shifted from language-related areas to visuospatial-related brain areas during mental calculation in association with the recovery of her mental abacus ability. Her subjective report was confirmed by the shift in brain activity. During digit memory tasks, activity in the bilateral superior parietal lobule and the right visual association cortex was observed after recovery. These results were consistent with the findings of previous research, as described in the literature reviewed above, suggesting that the right frontoparietal network plays an important role in mental calculation processing.

The brain is not a static structure. Substantial evidence shows that skill training can induce sustained, reproducible changes in brain activity and structure [38–42]. Structural methods such as voxel-based morphometry (VBM) on T1-weighted images and fiber tracking on diffusion tensor imaging (DTI) have been powerful tool for identifying when and where changes occur with learning in both gray matter (GM) and white matter (WM) [43]. VBM is one of the most widely used and best validated morphometric techniques for capturing structural alterations in the brain [44, 45]. This approach is very effective for shedding light on the patterns of learning/training-induced structural plasticity in the human brain [1]. A recent study by Li et al. [46] first investigated the impact of AMC training on brain structure. The authors used VBM to test whether structural changes could be detected in the brain of abacus-trained children as a result of extensive experience with AMC. The left fusiform gyrus (FG) of abacus-trained children was significantly smaller relative to those of average control subjects. Although the general observation is that measures of brain structure changes show the selective brain regions responsible for a specific skill [38, 40], some reports have recently demonstrated a decrease in GM [39]. The phenomenon that abacus-trained children showed a decrease of GM in the left FG may be taken as evidence of a reduction in GM. A functional neuroimaging study [4] showed that there was a number size effect in the left FG of an abacus-trained group during numeral mental-operation tasks. In addition to these functional and structural neuroimaging studies, neural pruning was used to explain the reduction in GM volume. Redundant or unused synapses were removed for neural pruning selection [2]. Fewer synapses were required to do the same amount of work; thus, differences between the groups were found in both functional and structural areas after training [43]. However, this result was based on children, and the sample size was relatively small. Future studies with large sample sizes of both children and adults are required to confirm this result.

Structural differences between abacus experts and nonexperts are not limited to GM as WM also appears to be susceptible to such effects [5, 46]. DTI and fiber tractography are new methods that can demonstrate the orientation and integrity of WM fibers in the brain* in vivo* [47–50]. A commonly used metric in DTI studies is factional anisotropy (FA), which quantifies the directional dependence of water diffusion in tissue [51, 52]. Group differences in brain WM were recently explored in children with over 3 years of training [5, 46]. Hu and colleagues reported that children with long-term AMC training have higher memory capacity and greater integrity in their WM tracts compared with nonexperts [5]. A tract-based spatial statistical approach was used in the above study to analyze DTI data collected after three years of AMC training. Compared with the nonexperts, increased FA values in the trained group were mainly located in the corpus callosum, the left occipitotemporal junction, and the right premotor projection. They concluded that the integrity of the WM tracts related to motor and visuospatial processes was different between the groups. This was the first DTI study of WM integrity in abacus-trained children. Subsequently, a fiber tracing method was used on the same subjects to explore the effect of training on the fiber pathway [46]. Fiber probability maps identified the left occipitotemporal junction as a key pathway connecting the left FG with other brain areas in the trained group, but not in the controls. Both studies were in good agreement with each other. The structural connectivity in children with long-term AMC training is significantly different compared with that of the nonexperts. However, whether this conclusion also holds for adult AMC experts needs to be studied further.

Based on the above VBM and DTI studies [5, 46], we can see that AMC training may have some effect on the brains of children, both at a macro- and at a microstructural level. These studies may improve our understanding of the structural plasticity underlying AMC training.

## 4. Limitations and Future Directions

Although the underlying cognitive and neural processing involved in abacus training has been well studied, there are multiple factors that can drive changes in cognitive function, brain activation, or structure. Much work remains regarding the effects of abacus training.

First, research designs need to be optimized. Currently, most research on AMC training uses cross-sectional designs in which subject responses are obtained at just one time-point. Using this method may lead to questions about whether differences are the consequence of physical or cognitive exercise. Preexisting differences between the groups were not considered in these studies, which may also have affected the results. The trained individuals may have advanced visuospatial ability that predisposed them to seek out and excel at AMC training. In addition, some nonexperts may also use a spatial strategy during mental calculations. There were some inconsistencies in the conclusions of these studies. To improve the accuracy of these conclusions, we advise using a longitudinal design [1, 53]. Preexisting individual differences would be determined before confounding experiences occurred. Cross-sectional studies can also misrepresent brain processes that occur within the individual. Longitudinal studies are elaborate and time-consuming but allow for the detection of subtle changes that cannot be detected in cross-sectional studies [53–57]. In the longitudinal design, the outcomes/responses of each subject are measured repeatedly, thereby allowing for the direct study of change over time. The only one longitudinal fMRI study on abacus training was designed by Tanaka et al. [35], wherein fMRI data were collected 6 and 13 months after the participant's stroke. Her brain activity shifted from language-related brain areas to visuospatial-related areas during mental calculation tasks, as her mental abacus ability recovered. However, this study was a case report. The sample sizes of all of the above reviewed neuroimaging studies were relatively small. Small sample sizes result in limited statistical power and limit meaningful conclusions. With the development of imaging techniques and advanced statistical analyses, sample sizes have increased. However, additional longitudinal studies with larger sample sizes are required to demonstrate the effect of AMC training on the function and structure of brain. Given the results of previous cross-sectional studies on abacus training, more cross-sectional neuroimaging research would be helpful for generating hypotheses for future investigations, whereas longitudinal research can be used to test and confirm these hypotheses. Furthermore, the authenticity of the research results can also be improved using a double-blinded and placebo-controlled design. Additionally, studies controlled training using other methods would lead to specific comparisons that explore the mechanism of AMC training.

Second, unimodal studies are still the predominant approach used to investigate brain changes or group differences after abacus training [3–5, 7, 36]. A single modality method may only partially detect potentially important variations in the brain. Combining multimodal imaging data would provide unprecedented opportunities to deepen our understanding of the existing cross-sectional data and may reveal the neural mechanisms involved in AMC training. There is increasing evidence that multimodal brain imaging studies can help provide a better understanding of the function-structure relationships and the functional or structural aspects of physiology that ultimately drive cognition and behavior [58–61]. Such approaches can provide a wealth of information, enabling researchers to more confidently draw conclusions. The most prevalent methods of functional imaging are fMRI and electroencephalography (EEG) [62], whereas structural MRI and DTI are the most common structural imaging modalities. Thus far, a multimodal method has been used in only two studies of abacus training, one using DTI-structural MRI [46] and another using fMRI-EEG [37]. Both studies are two-way fusion applications. Collecting data from three or more imaging modalities enables a more accurate understanding of brain networks and their relationship to human cognition and behavior [61, 63]. Combining multimodal data will be an important aspect of future abacus training research.

Third, most abacus studies focus on the effect of individual mathematical knowledge; few studies have focused on individual school achievement and cognitive development. Research on the transfer of abacus skills to other tasks will be important for understanding the effects of abacus training. Knowledge of the neural correlates of transfer may shed additional light on the underlying mechanisms [64]. Furthermore, whether abacus training has effects on real-life cognition (including the control of self-emotion, school achievement, social communication, and social cognition) will be another important trend of study of the training transfer effect. One way to address these research questions is to change the experimental environment of the controlled laboratory settings to more natural settings [65]. Another approach to dealing with these research questions could use a longitudinal study design, where participants are scanned repeatedly over time [53]. Once the training process is over, the question of whether training-induced brain plasticity and the cognitive ability are sustained can be addressed in longitudinal study. As we reviewed the effects of abacus training above, we found that abacus use may improve arithmetic abilities and attention in children. We think that this training may be applicable in both educational and clinical settings. For example, children with dyscalculia can be trained with the abacus practice to improve their arithmetic ability. Abacus training may be an option for children with psychiatric diagnoses. Long-term abacus training might also be useful as a nonpharmacological intervention to improve attention and alleviate problems underlying a range of disorders. This possibility should be explored in future investigations. In addition, the possible effects of abacus training on the neurocognitive domain should be evaluated more precisely.

Finally, the effect of abacus training on the functional connectivity of the brain remains largely unexplored. Functional connectivity is a statistical measure of correlation between functional MRI signals obtained from discrete brain regions [66–68]. A brief MRI data set acquired in resting subjects is sufficient to explore diverse brain systems. Although the functional connectivity between the visuospatial circuit and the attention circuit was enhanced in abacus-trained children [6], the connectivity between the training related network and other functional networks has not been explored. Future studies of this connectivity should be explored to further our understanding of the abacus training effect. The relationship between the functional and structural connectivity should also be studied in future research to explore the neural mechanism of abacus training. Furthermore, studies of the effects of abacus training on structural and functional connectivity were conducted in children only [5, 6, 46]; therefore, studies with adults should be a future endeavor.

## 5. Conclusion

In summary, we have conducted a brief review of the literature on the effects of abacus training, which has enabled us to draw a number of conclusions concerning the impact of training. The reviewed evidence shows that long-term AMC training affected both calculation skills and conceptual knowledge of the numeration system. Abacus training-induced functional and structural changes located mainly in the right frontoparietal network and the left FG areas. Future studies must verify these findings using better experimental designs, better analytical methods, and real-life outcomes, such as school achievement and cognitive development. The future challenge will be to understand the neural impact of this skill so that AMC may be translated into educational practice and treatment of people with mathematical disabilities.

## Glossary

AMC: Abacus-based mental calculation
DTI: Diffusion tensor imaging
EEG: Electroencephalography
fMRI: Functional magnetic resonance imaging
FA: Factional anisotropy
FG: Fusiform gyrus
GM: Gray matter
SPL: Superior parietal lobule
VBM: Voxel-based morphometry
WM: White matter.

## References

[1] May A. (2011). Experience-dependent structural plasticity in the adult human brain. *Trends in Cognitive Sciences*.

[2] Zatorre R. J., Fields R. D., Johansen-Berg H. (2012). Plasticity in gray and white: neuroimaging changes in brain structure during learning. *Nature Neuroscience*.

[3] Chen F., Hu Z., Zhao X. (2006). Neural correlates of serial abacus mental calculation in children: a functional MRI study. *Neuroscience Letters*.

[4] Hanakawa T., Honda M., Okada T., Fukuyama H., Shibasaki H. (2003). Neural correlates underlying mental calculation in abacus experts: a functional magnetic resonance imaging study. *NeuroImage*.

[5] Hu Y., Geng F., Tao L. (2011). Enhanced white matter tracts integrity in children with abacus training. *Human Brain Mapping*.

[6] Li Y., Hu Y., Zhao M., Wang Y., Huang J., Chen F. (2013). The neural pathway underlying a numerical working memory task in abacus-trained children and associated functional connectivity in the resting brain. *Brain Research*.

[7] Tanaka S., Michimata C., Kaminaga T., Honda M., Sadato N. (2002). Superior digit memory of abacus experts: an event-related functional MRI study. *NeuroReport*.

[8] Wang Y. Q., Geng F. J., Hu Y. Z., Du F. L., Chen F. Y. (2013). Numerical processing efficiency improved in experienced mental abacus children. *Cognition*.

[9] Bhaskaran M., Sengottaiyan A., Madhu S., Ranganathan V. (2006). Evaluation of memory in abacus learners. *Indian Journal of Physiology and Pharmacology*.

[10] Frank M. C., Barner D. (2012). Representing exact number visually using mental abacus. *Journal of Experimental Psychology: General*.

[11] Stigler J. W. (1984). ‘Mental abacus’: the effect of abacus training on Chinese children's mental calculation. *Cognitive Psychology*.

[12] Stigler J. W., Chalip L., Miller K. F. (1986). Consequences of skill: the case of abacus training in Taiwan. *American Journal of Education*.

[13] Hatano G., Miyake Y., Binks M. G. (1977). Performance of expert abacus operators. *Cognition*.

[14] Hishitani S. (1990). Imagery experts: how do expert abacus operators process imagery?. *Applied Cognitive Psychology*.

[15] Miller K. F., Stigler J. W. (1991). Meanings of skill: effects of abacus expertise on number representation. *Cognition and Instruction*.

[16] Hatano G., Osawa K. (1983). Digit memory of grand experts in abacus-derived mental calculation. *Cognition*.

[17] Hatta T., Miyazaki M. (1990). Visual imagery processing in Japanese abacus experts. *Imagination, Cognition and Personality*.

[18] Dehaene S., Spelke E., Pinel P., Stanescu R., Tsivkin S. (1999). Sources of mathematical thinking: behavioral and brain-imaging evidence. *Science*.

[19] Frank M. C., Everett D. L., Fedorenko E., Gibson E. (2008). Number as a cognitive technology: evidence from Pirahã language and cognition. *Cognition*.

[20] Hatano G., Amaiwa S., Shimizu K. (1987). Formation of a mental abacus for computation and its use as a memory device for digits: a developmental study. *Developmental Psychology*.

[21] Hatta T., Ikeda K. (1988). Hemispheric specialization of abacus experts in mental calculation: evidence from the results of time-sharing tasks. *Neuropsychologia*.

[22] Jaeggi S. M., Buschkuehl M., Jonides J., Shah P. (2011). Short-and long-term benefits of cognitive training. *Proceedings of the National Academy of Sciences of the United States of America*.

[23] Klingberg T. (2010). Training and plasticity of working memory. *Trends in Cognitive Sciences*.

[24] Irwing P., Hamza A., Khaleefa O., Lynn R. (2008). Effects of Abacus training on the intelligence of Sudanese children. *Personality and Individual Differences*.

[25] Shen H. (2006). Teaching mental abacus calculation to students with mental retardation. *Journal of the International Association of Special Education*.

[26] Na K.-S., Lee S. I., Park J.-H., Jung H.-Y., Ryu J.-H. (2015). Association between abacus training and improvement in response inhibition: a case-control study. *Clinical Psychopharmacology and Neuroscience*.

[27] Lustig C., Shah P., Seidler R., Reuter-Lorenz P. A. (2009). Aging, training, and the brain: a review and future directions. *Neuropsychology Review*.

[28] Bandettini P. A. (2012). Twenty years of functional MRI: the science and the stories. *NeuroImage*.

[29] Leopold D. A. (2010). Neuroscience: fMRI under the spotlight. *Nature*.

[30] Durston S., Casey B. J. (2006). What have we learned about cognitive development from neuroimaging?. *Neuropsychologia*.

[31] Wu T.-H., Chen C.-L., Huang Y.-H., Liu R.-S., Hsieh J.-C., Lee J. J. S. (2009). Effects of long-term practice and task complexity on brain activities when performing abacus-based mental calculations: a PET study. *European Journal of Nuclear Medicine and Molecular Imaging*.

[32] Wu T. H., Chen C. L., Liu R. S., Huang Y. H., Lee J. S. The computing brain: abacus-based mental calculation correlation between abacus experts and normal subjects in PET study.

[33] Hatta T., Hirose T., Ikeda K., Fukuhara H. (1989). Digit memory of soroban experts: evidence of utilization of mental imagery. *Applied Cognitive Psychology*.

[34] Chen C. L., Wu T. H., Cheng M. C. (2006). Prospective demonstration of brain plasticity after intensive abacus-based mental calculation training: an fMRI study. *Nuclear Instruments and Methods in Physics Research, Section A: Accelerators, Spectrometers, Detectors and Associated Equipment*.

[35] Tanaka S., Seki K., Hanakawa T. (2012). Abacus in the brain: a longitudinal functional MRI study of a skilled abacus user with a right hemispheric lesion. *Frontiers in Psychology*.

[36] Du F., Chen F., Li Y., Hu Y., Tian M., Zhang H. (2013). Abacus training modulates the neural correlates of exact and approximate calculations in chinese children: an FMRI study. *BioMed Research International*.

[37] Ku Y., Hong B., Zhou W., Bodner M., Zhou Y.-D. (2012). Sequential neural processes in abacus mental addition: an EEG and fMRI case study. *PLoS ONE*.

[38] Bezzola L., Mérillat S., Gaser C., Jäncke L. (2011). Training-induced neural plasticity in golf novices. *The Journal of Neuroscience*.

[39] Duan X., He S., Liao W. (2012). Reduced caudate volume and enhanced striatal-DMN integration in chess experts. *NeuroImage*.

[40] Hyde K. L., Lerch J., Norton A. (2009). Musical training shapes structural brain development. *The Journal of Neuroscience*.

[41] Ott U., Hölzel B. K., Vaitl D. (2011). Brain structure and meditation: how spiritual practice shapes the brain. *Neuroscience, Consciousness and Spirituality*.

[42] Tang Y.-Y., Tang R., Posner M. I. (2013). Brief meditation training induces smoking reduction. *Proceedings of the National Academy of Sciences of the United States of America*.

[43] Johansen-Berg H. (2012). The future of functionally-related structural change assessment. *NeuroImage*.

[44] Ashburner J., Friston K. J. (2000). Voxel-based morphometry—the methods. *NeuroImage*.

[45] Good C. D., Johnsrude I. S., Ashburner J., Henson R. N. A., Friston K. J., Frackowiak R. S. J. (2001). A voxel-based morphometric study of ageing in 465 normal adult human brains. *NeuroImage*.

[46] Li Y., Wang Y., Hu Y., Liang Y., Chen F. (2013). Structural changes in left fusiform areas and associated fiber connections in children with abacus training: evidence from morphometry and tractography. *Frontiers in Human Neuroscience*.

[47] Jbabdi S., Johansen-Berg H. (2011). Tractography: where do we go from here?. *Brain Connectivity*.

[48] Johansen-Berg H., Rushworth M. F. S. (2009). Using diffusion imaging to study human connectional anatomy. *Annual Review of Neuroscience*.

[49] Le Bihan D. (2003). Looking into the functional architecture of the brain with diffusion MRI. *Nature Reviews Neuroscience*.

[50] Le Bihan D., Johansen-Berg H. (2012). Diffusion MRI at 25: exploring brain tissue structure and function. *NeuroImage*.

[51] Basser P. J., Pierpaoli C. (1996). Microstructural and physiological features of tissues elucidated by quantitative-diffusion-tensor MRI. *Journal of Magnetic Resonance—Series B*.

[52] Scholz J., Klein M. C., Behrens T. E. J., Johansen-Berg H. (2009). Training induces changes in white-matter architecture. *Nature Neuroscience*.

[53] Skup M. (2010). Longitudinal fMRI analysis: a review of methods. *Statistics and Its Interface*.

[54] Engvig A., Fjell A. M., Westlye L. T. (2012). Memory training impacts short-term changes in aging white matter: a Longitudinal Diffusion Tensor Imaging Study. *Human Brain Mapping*.

[55] Giorgio A., Watkins K. E., Chadwick M. (2010). Longitudinal changes in grey and white matter during adolescence. *NeuroImage*.

[56] Jiang J., Sachdev P., Lipnicki D. M. (2014). A longitudinal study of brain atrophy over two years in community-dwelling older individuals. *NeuroImage*.

[57] Szaflarski J. P., Schmithorst V. J., Altaye M. (2006). A longitudinal functional magnetic resonance imaging study of language development in children 5 to 11 years old. *Annals of Neurology*.

[58] Damoiseaux J. S., Greicius M. D. (2009). Greater than the sum of its parts: a review of studies combining structural connectivity and resting-state functional connectivity. *Brain Structure and Function*.

[59] Rykhlevskaia E., Uddin L. Q., Kondos L., Menon V. (2009). Neuroanatomical correlates of developmental dyscalculia: combined evidence from morphometry and tractography. *Frontiers in Human Neuroscience*.

[60] Sui J., Adali T., Yu Q., Chen J., Calhoun V. D. (2012). A review of multivariate methods for multimodal fusion of brain imaging data. *Journal of Neuroscience Methods*.

[61] Sui J., Huster R., Yu Q., Segall J. M., Calhoun V. D. (2014). Function-structure associations of the brain: evidence from multimodal connectivity and covariance studies. *NeuroImage*.

[62] Michel C. M., Murray M. M. (2012). Towards the utilization of EEG as a brain imaging tool. *NeuroImage*.

[63] Smith S. M. (2012). The future of FMRI connectivity. *NeuroImage*.

[64] Dahlin E., Bäckman L., Neely A. S., Nyberg L. (2009). Training of the executive component of working memory: subcortical areas mediate transfer effects. *Restorative Neurology and Neuroscience*.

[65] Hasson U., Honey C. J. (2012). Future trends in neuroimaging: neural processes as expressed within real-life contexts. *NeuroImage*.

[66] Buckner R. L., Krienen F. M., Yeo B. T. T. (2013). Opportunities and limitations of intrinsic functional connectivity MRI. *Nature Neuroscience*.

[67] Uddin L. Q. (2013). Complex relationships between structural and functional brain connectivity. *Trends in Cognitive Sciences*.

[68] Van Den Heuvel M. P., Hulshoff Pol H. E. (2010). Exploring the brain network: a review on resting-state fMRI functional connectivity. *European Neuropsychopharmacology*.

